# Single-Center Analysis of Human Papillomavirus Infection and P16INK4A Expression among Korean Patients with Penile Cancer

**DOI:** 10.1155/2019/6940582

**Published:** 2019-03-26

**Authors:** Whi-An Kwon, Min Young Yoon, Sung Han Kim, Ho Kyung Seo, Jinsoo Chung, Kang Hyun Lee, Jae Young Joung, Weon Seo Park

**Affiliations:** Center for Prostate Cancer, National Cancer Center, Goyang, Republic of Korea

## Abstract

**Purpose:**

This study aimed to evaluate the statuses of P16INK4A expression and human papillomavirus (HPV) infection among patients with penile cancer at a single Korean institution.

**Patients and Methods:**

Fourteen patients with penile cancer at our center were retrospectively identified and their clinicopathological data were analyzed. The patients' HPV and P16INK4A expression status (a known tumor suppressor protein) were tested using genotyping with a DNA chip assay and immunohistochemical staining, respectively. The results regarding HPV status were compared to those from another Asian study.

**Results:**

The mean age at diagnosis was 60 years (range: 34–86 years). The median tumor size was 3.0 cm (range: 0.6–4.7 cm). Ten tumors were located on the penile glans. Five patients tested positive for HPV DNA (5/14, 36%) and all cases involved HPV type 16 (5/5, 100%). Positive expression of P16INK4A was observed in 6 cases (6/14, 43%). Among the HPV positive cases, 80% of cases (4/5) were also positive for P16INK4A. The prevalence of HPV infection in our study (36%) was higher than in a previous Asian study (23%).

**Conclusions:**

This is the first study to evaluate the prevalence of HPV infection and P16INK4A expression among patients with penile cancer at a single Korean institution. The prevalence of HPV (36%) was slightly higher than the results from a previous Asian study. Additional multi-center studies are needed to better understand penile cancer in Korea and to identify biomarkers that can determine high-risk cases and predict their prognosis.

## 1. Introduction

Penile cancer is a rare disease that is associated with high rates of morbidity and mortality when it has progressed [1]. Squamous cell carcinoma (SCC) is the most prevalent histological type in developing countries, such as Asia, Africa, and South America [2, 3]. The overall incidence in the US is approximately 0.69 cases/100,000 people, although this rate increases with an increasing age at diagnosis [4]. In Korea, primary penile cancer is both rare and rarely reported, accounting for only 74 cases in 2015, which corresponded to only 0.03% of all malignant tumors during that year [5].

Previous studies have described two major pathways for penile cancer development. The first involves various penile conditions, such as phimosis, inflammation, and a history of lichen sclerosus or lichen planus. The second involves human papillomavirus (HPV) infection [2, 6], with HPV DNA being detectable in approximately one-half of penile cancer cases [7–9]. The most common type is HPV-16, which is followed by HPV-18 [8]. To determine whether a positive result for HPV DNA in tumor tissue is related to active infection, transient infection, or contamination, researchers evaluate various markers for HPV activity and induced carcinogenesis, such as viral transcription and HPV-induced cell mutation markers [10]. These markers can guide the prediction of the patient's prognosis, based on the etiology and virulence status of the penile cancer.

p16INK4a (also known as p16INK4a^INK4a^, cyclin-dependent kinase inhibitor 2A, multiple tumor suppressor 1 and as several other synonyms), is a tumor suppressor protein, that in humans is encoded by the CDKN2A gene [11]. Recently, many studies have reported that expression of the p16INK4a^INK4a^ protein was significantly associated with the presence of HR-HPV [12–14]. However, no Korean studies have evaluated the association between HPV and penile cancer, and this single-center study aimed to provide preliminary data regarding the prevalence of HPV infection and P16INK4A expression (a known tumor suppressor) among Korean patients with penile cancer.

## 2. Materials and Methods

### 2.1. Patients and Materials

This retrospective study's protocol was approved by our institutional ethics review board (NCCNCS08200). Medical records were obtained for 17 consecutive patients who underwent surgery for penile cancer at the National Cancer Center between August 2012 and March 2018. The patients were followed up every 3 months for the first year and then every 6 months if they had not experienced tumor recurrence. After obtaining informed consent, paraffin-embedded specimens from these patients were prepared and stored.

The present study's inclusion criterion was surgically resected and histologically confirmed penile cancer, regardless of whether the patient underwent postoperative chemotherapy or radiotherapy. The exclusion criteria were contraindications for surgery-based treatments, early presentation with distant metastases, failure to complete the planned treatment course, or cases where the formalin-fixed paraffin-embedded samples are not available to test for P16INK4A and HPV status. The patients' clinical data were retrieved from their records and the tumors were staged based on the American Cancer Staging Standards Committee system from 2016. Histological grading was performed by pathologists according to the World Health Organization criteria for penile cancer. Our patients' HPV status was compared to that from another Asian study [15].

### 2.2. DNA Extraction

The DNA extraction was performed using the QIAamp Mag Attract DNA Mini M48 Kit (Qiagen, Valencia, CA) and the Qiagen BioRobot M48 workstation. The carcinoma area from the paraffin block was retrieved to obtain the greatest proportion of the tumor, which was then collected in 1.5-mL Eppendorf tubes for DNA extraction. A total of 10* μ*L of the purified total cellular DNA was used for each HPV PCR reaction.

### 2.3. HPV Genotyping Using an HPV Chip

The presence of HPV DNA was simultaneously tested based on genotyping using a PCR-based HPV DNA chip (Greencross, Gyeonggi, Korea), as previously described [16]. This chip is capable of identifying 15 types of high-risk HPV (HPV-16, HPV-18, HPV-31, HPV-33, HPV-35, HPV-39, HPV-45, HPV-51, HPV-52, HPV-53, HPV-56, HPV-58, HPV-59, HPV-66, and HPV-68) and 9 types of low-risk HPV (HPV-6, HPV-11, HPV-34, HPV-40, HPV-42, HPV-43, HPV-44, HPV-54, and HPV-70).

## 3. Results

The patients' main characteristics and human papillomavirus status are shown in Table 1. The mean age at diagnosis was 60 years (range: 34–86 years). Among the 14 patients, HPV was detected in 5 cases (36%) and all cases involved HPV type 16 (5/5, 100%). Expression of P16INK4A was detected in 6 cases (43%). The prevalence of HPV in our study (36%) was slightly higher than in a previous Asian study (23%) [15]. A comparison of the clinicopathological characteristics between our study and the previous Asian study is shown in Table 2.

The median tumor size was 3.0 cm (range: 0.6–4.7 cm). Ten tumors were located on the penile glans and the remaining 4 tumors were located on the shaft. All tumors were invasive (pT1: tumour invades subepithelial connective tissue–pT3: tumour invades corpus cavornosum). Five patients had lymph node invasion at the time of diagnosis (N1-2) or developed metastatic lymph nodes during follow-up. None of the patients had distant metastases. Eight tumors were well differentiated (G1) and 6 tumors were moderately differentiated (G2). All patients underwent total or partial penectomy, and 6 patients also underwent concurrent lymphadenectomy. The median follow-up was 54 months (range: 1–127 months), and a review of the records revealed that 3 patients died and 5 patients were known to be alive at the time of the analysis.

### 3.1. Immunostaining and Scoring

**Figure 1:**
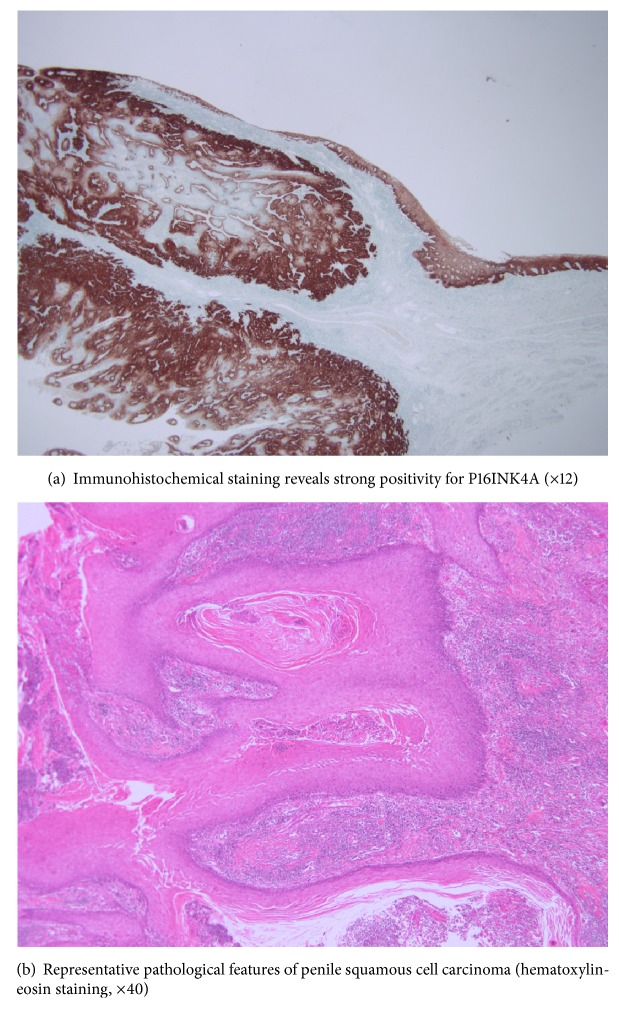
Representative findings for squamous cell carcinoma and P16INK4A expressions.

Immunohistochemical staining revealed strong positivity for P16 (×12) (Figure 1(a)). Immunohistochemistry was performed to evaluate P16INK4A expression (1:50, P2D11F11; Novocastra, Buffalo Grove, IL), with the results categorized as negative (subjective score: 0) or positive (subjective score 1–3). Representative pathological features of penile squamous cell carcinoma were shown in Figure 1(b). An experienced urologic pathologist (WSP) was blinded to the patients' clinical data before performing the expression scoring and evaluating the general pathological characteristics. We used a control for p16INK4a expression and HPV detection. Expression of P16INK4A was strongly positive in nucleus and cytoplasm.

## 4. Discussion

To the best of our knowledge, this is the first study to evaluate the characteristics, HPV status, and P16INK4A expression among patients who underwent surgery for penile cancer at a single Korean institution. The mean patient age at the diagnosis in the present study was 60.4 years, which is similar to the age of 62 years that was reported by Chaux et al. [17].

Penile cancer is known to be associated with lack of circumcision, HPV infection, phimosis, smoking, and poor hygiene, with HPV DNA being detectable in 70–100% of penile intraepithelial neoplasia and approximately 40% of penile cancers being positive for HPV [2]. In the present study, HPV was detected in 36% of cases (5/14), which is similar to or lower than the reported rates of 33% by Alemany et al. [18], 47% by Miralles-Guri et al. [8], and 50% by Backers et al. [7]. However, the prevalence of HPV in our study was slightly higher than in a previous Asian study (23%) [15]. These differences may be related to the small sample sizes and the variety of detection methods, as well as differences in HPV exposure and other factors (e.g., the prevalence of circumcision, a protective factor).

HPV prevalence for penile invasive cancer in our study (36%) is lower than those found in Miralles-Guri et al. (47%) [8], Backes et al. (50%) [7], or a 2014 study of penile cancers by Hernandez et al. (63%) [19]. The observed differences might be due to variation in small studies using various detection methods included in a systematic review. Furthermore, differential case selections of geographical origins and reporting biases of histology might also explain this difference.

P16INK4A has presently been used as an alternative marker of high-oncogenic risk HPV infection in cervical and other carcinomas [12], and some studies have shown that p16INK4a overexpression in penile cancers is associated with HPV infection [20, 21]. However, other studies have shown different results [22, 23]. Recently, Vicenilma et al. [12] have reported that expression of the p16INK4a was significantly associated with the presence of high-oncogenic risk HPV and this expression may serve as a marker for the presence of the virus in penile cancer. Our study showed that positive expression of P16INK4A was observed in 6 cases (6/14, 43%). A possible explanation for HPV/p16INK4a discordance of this study is that the HPV detection method was not able to detect HPV, even though it was present.

Most of our patients were smokers (86%), and there is a clear dose-response relationship between smoking and the development of penile cancer. For example, Daling et al. have reported that patients who smoked >10 cigarettes per day had a significantly higher risk of developing penile cancer than patients who smoked lesser amounts [24]. Koifman et al. [25] also reported that the incidence of smoking among penile cancer patients was high (56.5%). Nevertheless, the precise role of smoking in the development and progression of penile cancer remains unclear.

In Korea, the incidence of penile cancer is 0.1 cases/100,000 men, which ranks along with Israel as one of the lowest incidences in the world. This is likely related to the fact that the vast majority of Korean men are circumcised [5, 26], although this relationship may be related to circumcision protecting against the development of phimosis [2]. Hellberg et al. [27] have related that the relative risk of penile cancer was 64.6 among men with phimosis and that phimosis was a risk factor for tumor development [2, 24]. Morris, B. J. et al. [26] emphasized that penile cancer is strongly associated with lack of circumcision and also presented meta-analyses of association of penile cancer with phimosis and balanitis. Furthermore Morris, B. J. et al [28] showed that circumcised males have a 68% lower prevalence of balanitis than uncircumcised males and that balanitis is accompanied by a 3.8-fold increase in risk of penile cancer. Nevertheless, circumcision has recently become less common in Korea, and it may be relevant to emphasize the relationship between circumcision and a decreased incidence of penile cancer.

The present study has several limitations. First, the study used an uncontrolled retrospective design, without a central decision-making strategy and standardized surgical techniques. Second, the study evaluated a small sample of patients at a single center, which is linked to issues with the statistical analysis and reliability of the findings. Therefore, additional studies are needed to evaluate this issue in a larger sample of patients who are treated in multiple centers.

## 5. Conclusion

To the best of our knowledge, this is the first study to evaluate the prevalence of HPV infection and P16INK4A expression among patients who underwent surgery for penile cancer at a single Korean center. The prevalence of HPV was 36%, which was slightly higher than the rate from another Asian study. In Korea, penile cancer is relatively rare in the general population, which has led to few studies to examine its characteristics and propose a codified approach to treating these patients. We hope that this study will highlight the need for multi-center studies to better understand the prevalence and characteristics of penile cancer in Korea.

## Figures and Tables

**Table 1:** The patients' main characteristics and human papillomavirus status.

	P1	P2	P3	P4	P5	P6	P7	P8	P9	P10	P11	P12	P13	P14
Age at diagnosis	34	75	49	51	48	70	78	62	80	42	86	71	49	50

P16INK4A status	F	N	N	P	F	N	P	N	P	P	P	P	N	N

HPV status	N	N	N	16	16	N	16	N	N	16	N	16	N	N

Smoking	Current	Current	Current	Current	Current	Former	Former	Current	Former	Current	Former	Never	Former	Never

Location	Glans	Glans	Glans	Penile shaft	Penile shaft	Glans	Glans	Glans	Glans	Penile shaft	Glans	Penile shaft	Glans	Glans

Size	2.0 cm	4.0 cm	3.0 cm	4.0 cm	3.0 cm	3.0 cm	1.6 cm	3.5 cm	2.2 cm	0.6 cm	3.0 cm	4.7 cm	2.4 cm	2.5 cm

pT	T3	T2	T3	T3	T1a	T1b	T3	T1a	T1a	T1a	T1a	T3	T2	T1a

pN	Nx	N0	N0	N1	N0	N0	N1	N1	Nx	Nx	N0	N2	N2	Nx

pM	M0	M0	M0	M0	M0	M0	M0	M0	M0	M0	M0	M0	M0	M0

Grade	1	1	1	2	1	2	2	1	1	2	1	2	2	1

AJCC stage	2	2	3	3	1	2	3	3	1	1	1	3	3	1

Type	SCC	SCC	SCC	SCC	SCC	SCC	SCC	SCC	SCC	SCC	SCC	SCC	SCC	SCC

Treatment	Partial penectomy	Partial penectomy	Total penectomy	Total penectomy	Partial penectomy + lymphadectomy	Partial penectomy	Total penectomy + lymphadenectomy	Partial penectomy + lymphadectomy	Partial penectomy	Total penectomy	Partial penectomy + lymphadectomy	Total penectomy + lymphadenectomy	Total penectomy + lymphadenectomy	Partial penectomy

Resection margin	-	-	-	+	-	+	-	-	-	-	-	-	-	-

Follow-up, months	120	NA	94	14	127	4	NA	84	9	95	NA	5	54	1

Death	NA	Yes	NA	NA	No	NA	Yes	No	NA	No	Yes	NA	No	No

OS, months	NA	143	NA	NA	NA	NA	11	NA	NA	NA	20	NA	NA	NA

HPV: human papillomavirus, N: negative, F: false, P: positive, SCC: squamous cell carcinoma, Lt: left, LN: lymph node, NA: not available.

**Table 2:** Comparing the clinicopathological characteristics between our study and a previous Asian study.

	Our study (N = 14)	A previous Asian study [15] (N = 120)
HPV-positive status, n (%)	5/14 (36%)	27/120 (23%)

Mean age at diagnosis, years	60.4	53.0

Histological grade		
1	8 (57%)	61 (51%)
2	6 (43%)	36 (30%)
3		19 (16%)
Undetermined	0 (0%)	4 (3%)

Stage		
1	5 (36%)	1 (1%)
2	3 (21%)	29 (24%)
3	6 (43%)	23 (19%)
4	0 (0%)	6 (5%)
Unknown	0 (0%)	61 (51%)

Smoking history		
No	2 (14%)	44 (37%)
Yes	12 (86%)	12 (10%)
Unknown	0 (0%)	64 (53%)

HPV: human papillomavirus.

## Data Availability

The data used to support the findings of this study are available from the corresponding author upon request.

## Abbreviations

SCC: Squamous cell carcinoma
HPV: Human papillomavirus.
